# Molecular biomarkers NOTCH1, CD44, BMI1, and TP53 in oral squamous cell carcinoma

**DOI:** 10.6026/97320630019523

**Published:** 2023-05-31

**Authors:** Mohammad Reshma, Roy Anitha, Rajagopalan Vijayaraghavan, S. Sreelatha, Sarvari Geriki

**Affiliations:** 1 a Department of Research and Develpoment, Saveetha Institute of Medical and Technical Sciences, Thandalam, Chennai, Tamil Nādu; 2 Department of Pharmacology, Saveetha Dental College and Hospitals, Saveetha Institute of Medical and Technical Sciences, Chennai, Tamil Nadu; 3 Department of Anatomy, Malla Reddy Medical College for Women, Hyderabad, Telangana; 4 Department of Biochemistry, ACSR Government Medical College, SPSR, Nellore

**Keywords:** NOTCH1, CD44, BMI1, TP53, human papillomavirus and oral malignancies

## Abstract

It is of interest to evaluate NOTCH1, CD44, BMI1, and TP53 genes in the epiglottis, tongue, and hard palate of oral malignancies (OM) with healthy controls. This was a prospective and cross-sectional study of 60 individuals with oral malignancies (OM) (20 each of tongue, epiglottis, and hard palate) studied at Malla Reddy Medical College and tertiary care hospitals in Hyderabad. Adults aged ≥ 18 years and diagnosed with oral cancer were included in the study. Those who had cancer in more than one area were excluded from the study. Blood samples of individuals with tongue or epiglottis or hard palate were taken for testing the expression of NOTCH1, CD44, TP53, and BMI1 genes. They were analysed by the genomic sequencing method. One-way ANOVA with Bonferroni's t-test was used for statistical analysis. Expression of NOTCH1, CD44, BMI1, and TP53 genes were significantly higher in epiglottis, tongue, and hard palate compared to healthy control samples (p < 0.001). All four genes were expressed in all three areas of OM. However, they were not significant between them. Further analysis revealed that NOTCH1, CD44, TP53, and BMI1 genes did not show any difference in HPV-positive and HPV-negative samples. Comparing the T stages of cancer Notch1, gene expression was significantly higher in stages 1 and 2 compared to 3 and 4. The CD44, TP53, and BMI1 did not show any differences in the T stage. However, the difference in HPV in all T stages was very minimal. Data showed that irrespective of the areas of cancer (epiglottis, tongue, and hard palate) NOTCH1, CD44, TP53, and BMI1 genes were expressed equally. The expression was not very much dependent on HPV positive (+ve) or negative (-ve). However the T-stage was showing higher expression compared to control group. Since the expression of these genes was very high in all the three malignancies, they may be used as early biomarkers to detect cancer of epiglottis, tongue, and hard palate.

## Background:

Cancer is a large group of diseases and can start in any organ or tissue when abnormal cells grow uncontrollably. They can invade adjoining parts of the body and spread to other organs. [1] Head and neck squamous cell carcinomas (HNSCC) constitute a heterogeneous group of cancers, which include cancers arising in the oral cavity, nasopharynx, oropharynx, hypopharynx, and larynx. [2] Worldwide, head and neck cancer accounts for approximately 900,000 cases and over 400,000 deaths annually. In the United States, head and neck cancer accounts for 3 percent of malignancies, and causes about 15,000 deaths annually. [3] Men are affected more than women, with a ratio ranging from 2:1 to 4:1. The incidence rate in men exceeds 20 per 100,000 in regions of France, Hong Kong, and the Indian subcontinent. [4] HNSCC is the sixth most common cancer worldwide, with 890,000 new and 450,000 deaths in 2018. The incidence of HNSCC continues to rise and is anticipated to increase by 30%. [5] The molecular pathology of HNSCC has been studied extensively and common somatic genetic changes have been reported. [6] There are studies of the multistage evolution of the tumors but are not characterized. A small number of tumour arise from pre-existing lesions, known as potentially malignant lesions (PPOL) such as leucoplakia or erythroplakia, which display variable epithelial dysplasia. [7] Particularly, HNSCC was characterized by mutation of TP53, genome duplications, multiple recurrent chromosomal gains, and increased genomic disruption affecting cell cycle checkpoints and PI3K-AKT signaling. [8] Increased rates of somatic copy number alterations (SCNAs) of the tumour genome were associated with poor prognosis. It is important to identify SCNAs that might be functionally driving progression and outcome. In addition, genomic studies have revealed how differential genomic patterns among cases could identify various subgroups of tumours. Histological subtypes, smoking, and human papillomavirus (HPV) status may alter the disease progression. [9] Genomic profiling studies are important to understand molecular abnormalities in HNSCC for the development of new therapeutics. Therefore, it is of interest to evaluate NOTCH1, CD44, BMI1, and TP53 genes in the epiglottis, tongue, and hard palate and compare them with healthy controls.

## Material and Methods:

This study was a prospective and cross-sectional study of oral squamous cell carcinoma. 60 individuals (20 of tongue; 20 of the epiglottis; 20 of the hard palate) from Malla Reddy Cancer Hospital and Research Institute and Tertiary Care Hospitals (Hyderabad, India) were the participants. Adults aged ≥ 18 years both male and female diagnosed with oral squamous cell carcinoma, oropharynx, nasopharynx, larynx, hypopharynx of head and neck origin and histologically confirmed were included. Individuals with parotid tumours, previous radiotherapy, chemotherapy, and heavy tumour bleeding from the mouth were excluded. The present study was approved by the MRMCW Institute Ethics committee (Project No. MRMCWIEC/AP/80/2022). Information about the research study was given in English and Telugu (regional language) and signed consent was obtained. Confidentiality of the information was maintained. Control samples from 17 normal individuals were also taken.

## Methodology:

3mL - 5mL of blood sample was collected from each participant and plasma was separated. DNA was extracted by genomic DNA isolation kit (Thermofisher scientific K0512 Genomic DNA Purification Kit). DNA quality was checked by UV absorption at 260 and 280 nm by agarose gel electrophoresis. The isolated DNA was stored at 4°C for genetic analysis. PCR was used to amplify the NOTCH1, TP53, CD44, and BMI1 genes. Cycling conditions were 90°C for 5 min (1 cycle) 90°C for 40 sec, 55°C for 40 sec, and 72°C for 60 sec. Final extension at 72°C for 10 min by 1 pair of primers. 3 sets of primers were designed for each gene. Mutation analysis was done for all four genes. Nucleotide sequences of all amplified PCR were determined in both orientations by sequencing with an applied Bio-Systems 3730 XL sequence. The result was analyzed using Bio E, dit (V7.1.3). Sequence scanner (V 1.0) Applied Biosystems (0) and Nucleotide BLAST programs.

## Statistical analysis:

The data were expressed as mean and standard error (mean ± SE). The means were compared by student's 't' 5. test or one-way analysis of variables with Bonferroni's t-test. SigmaPlot 14.5 version (Systat]8 Software Inc., San Jose, USA) was used for the statistical analysis and graph plotting. A p-value of less than 0.05 was taken as statistically significant.

## Result

**Figure 1:**
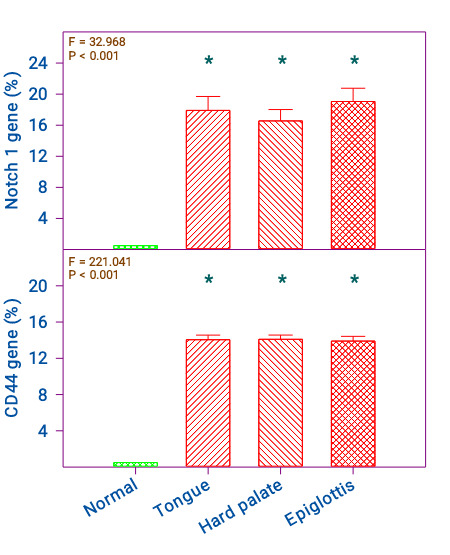
Comparison of normal, and cancer of the tongue, hard palate, and epiglottis on NOTCH 1 and CD 44 genes. n = Normal = 17; Tongue, Hard palate, and Epiglottis 20 each. The 'F' and 'P' values were by one-way ANOVA with Bonferroni's t-test. *Significantly different from normal.

**Figure 2:**
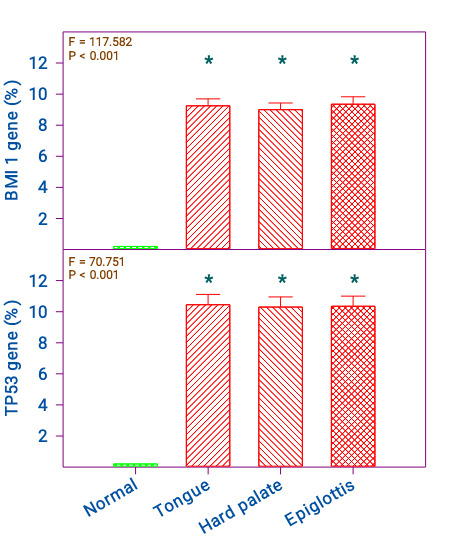
Comparison of normal and cancer of the tongue, hard palate, and epiglottis on TP53 and BMI1 genes; n = Normal = 17; Tongue, Hard palate, and Epiglottis 20 each. The 'F' and 'P' values were by one-way ANOVA with Bonferroni's t-test.

**Figure 3:**
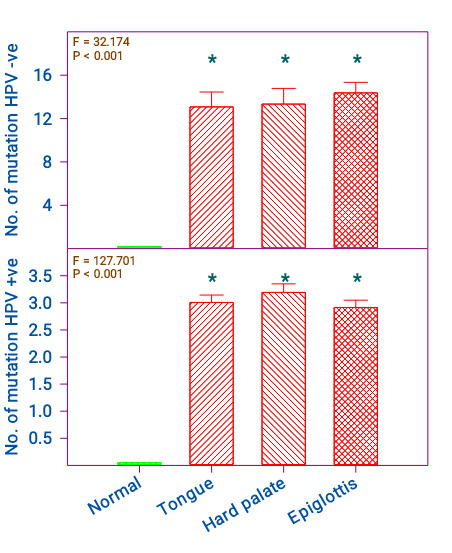
Comparison of normal, and cancer of the tongue, hard palate, and epiglottis in HPV +ve and -ve. n = Normal = 17; Tongue, Hard palate, and Epiglottis 20 each. The 'F' and 'P' values were by one-way ANOVA with Bonferroni's t-test. *Significantly different from normal.

The expression of the Notch1 gene in tongue, epiglottis, and hard palate malignancies was 17.9 ± 1.8, 16.6 ± 1.5, and 19.1 ± 1.7(%) respectively and was significant compared to control (p < 0.001). The control samples did not show any expression. All three malignancies in tongue, epiglottis, and hard palate respectively showed similar expression and did not show any significance between them (Figure 1). The expression of the CD44 gene in tongue, epiglottis, and hard palate malignancies was 14.1 ± 2.3, 14.1 ± 2.1, and 13.9 ± 2.3 (%) respectively and was significant compared to control (p < 0.001); however, they did not show any significance between them (Figure 1). The expression of the BMI1 gene in tongue, epiglottis, and hard palate malignancies were 9.3 ± 2.0, 9.0 ± 1.9 and 9.4 ± 2.1(%) respectively and was significant compared to control (p < 0.001). However, they did not show any significance between them. The control samples did not show any expression (Figure 2). The expression of the TP53 gene in the tongue, epiglottis, and hard palate were 10.5 ± 3.0, 10.3 ± 2.9 and 10.4 ± 2.9 (%) respectively. The control samples did not show any expression. Hence, the gene expression was significant compared to control (p < 0.001) and between them, they were not significant (Figure 3).

The number of mutations in HPV +ve and -ve samples also showed similar findings. The number of mutations in HPV +ve samples in the tongue, epiglottis and hard palate were 13.1 ± 1.4, 13.3 ± 1.4, and 14.4 ± 1.0 respectively. The control samples showed 0.2 ± 0.0 (p < 0.001). The tongue, epiglottis and hard palate showed similar pattern. However, between them they were not significant (Figure 3). The number of mutations in the HPV -ve samples in the tongue, epiglottis, and hard palate was 3.0 ± 0.1, 3.3 ± 0.2, and 2.9 ± 0.1 respectively. The control samples showed 0.1 ± 0.0 and were significant compared to control (p < 0.001). All three regions showed an equal number of mutations and between them, they were not significant (Figure 3).

The analysis of Notch1, CD44, BMI1, and TP53 genes in HPV +ve and -ve samples also showed that the difference among them was very minimal (Table 1). The analysis of all the four genes in 'T' stages 1-2 and 3-4 also revealed that the difference among them was not significant. Though they were highly expressed compared to the control (p < 0.001) (Table 2).

## Discussion:

Head and neck cancer is a broad term that encompasses epithelial malignancies which are squamous cell carcinoma of the head and neck (SCCHN). The most risk factors for these carcinomas were tobacco and alcohol consumption which account for higher rates of SCCHN. Evidence also suggests that human papillomavirus (HPV) is a cause of specific subsets of SCCHN [9]. According to Chaturvedi et al., HPV is an important cause of oral squamous cell carcinoma (OSCC). It is currently unclear whether HPV may also have a role in other head and neck cancer sub-sites, including (OSCC) [10]. Thus, the persistence of the virus might be a critical factor in the development of HPV-related diseases [11]. The present study agreed with the studies of Chaturvedi et al. and Zhang. et al., that number of mutations in HPV +ve and -ve samples also showed similar findings. The tongue, epiglottis, and the hard palate showed an equal number of mutations and mutually they were not significant. Patients with HPV positive have distinct clinical features from HPV negative. HPV positivity is a favourable prognostic factor and has a better response towards radiation, chemotherapy, or both than HPV-negative tumours. Thus, the persistence of the virus might be a critical factor in the development of HPV-related diseases [12]. Brazil and Argentina could contribute to the low percentage of HPV-positive HNSCC, as has been previously described in South America [13]. In contrast to the clear picture in OPSCC, where the prognostic relevance of biologically active HPV infection is established, no such clear association can be found for OSCC. Several publications analysing larger cohorts underline that HPV DNA and especially HPV16 is present in 10-25% of tumours of the oral cavity, which is higher than in the healthy control population but smaller than in OPSCC [14]. Molecular changes in HNSCC may potentially encode key signalling molecules like TP53, NOTCH1, CDKN2A, PIK3CA, HRAS, and PTEN genes [15]. However, no statistically significant differences were detected in the age and gender concerning NOTCH1 mutations in T-cell acute lymphoblastic leukaemia (T-ALL) patients [16]. Another finding also showed similar results [17]. The NOTCH1 mutations in T-ALL did not differ according to gender or age. This result was in agreement with the finding reported by Fogelstrand et al., Baldus et al., [15 and 18]. In the present study Notch 1, CD44, BMI1, and TP53 gene mutations were studied in different regions of oral cancer. The expression of Notch1, CD44, TP53, and BMI1 genes in tongue, epiglottis, and hard palate malignancies was similar. In a study, the majority of gene expressions in HNSCC were identified in advanced stages [19]. However, in the present study the genes (NOTCH1, CD44, BMI1, and TP53) were significantly expressed in T-stages, 1-2 and 3-4 of cancer showing that in earlier stages also they can be highly expressed; similarly in the present study both HPV positive and HPV negative cases did not show any significant difference in all the genes studied, showing that HPV may not be expressed for the OSCC. Early-stage detection increases survival rates compared with late-stage disease. Hence, NOTCH1, CD44, BMI1, and TP53 can be routinely monitored in suspected cases for early detection.

## Conclusion:

The present study revealed that NOTCH1, CD44, BMI1, and TP53 are expressed several folds compared to healthy control and the expression was not dependent on gender, age and HPV. They were significantly expressed in T-stages, 1-2 and 3-4 of cancer showing that in earlier stages also they can be highly expressed. Hence, these genes can be routinely monitored in suspected cases of malignancies for early detection and treatment.

## Funding Source:

Self

## Figures and Tables

**Table 1:** Comparison of Notch1, CD44, BMI 1 & TP53 genes in HPV Positive and Negative cases

**S.No.**	**Variable**	**HPV**	**Mean**	**SE**	**Statistics**
1	Notch 1 gene (%)	+ve	18.95	1.35	t = 1.495 P = 0.140
		-ve	16.04	1.15	
2	CD44 gene (%)	+ve	13.97	0.36	t = 0.193 P = 0.848
		-ve	14.09	0.47	
3	BMI 1 gene (%)	+ve	9.22	0.3	t = 0.0794 P = 0.937
		-ve	9.17	0.47	
4	TP53 gene (%)	+ve	10.16	0.51	t = 0.694 P = 0.490
		-ve	10.7	0.52	
n = HPV +ve = 37; HPV -ve = 23

**Table 2:** Comparison of T stages of cancer of the tongue, hard palate, and epiglottis in Notch1, CD44, BMI 1, TP53.

**Variable**	**T stage**	**Mean**	**SE**	**Statistics**
Notch 1 gene (%)	0	0	0	F = 48.871 P < 0.001
	1 and 2	18.04*	1.01	
	3 and 4	16.50*	3.03	
CD44 gene (%)	0	0	0	F =336.394 P < 0.001
	1 and 2	14.05*	0.31	
	3 and 4	13.75*	0.64	
BMI 1 gene (%)	0	0	0	F =181.825 P < 0.001
	1 and 2	9.30*	1.97	
	3 and 4	8.50*	2.07	
TP53 gene (%)	0	0	0	
	1 and 2	10.26*	0.41	
	3 and 4	11.00*	0.73	
n = Total = 17; T1 and T 2 = 52; T3 and T4 = 8. *Significantly different from T stage 0.
